# The Megalocytivirus RBIV Induces Apoptosis and MHC Class I Presentation in Rock Bream *(Oplegnathus fasciatus)* Red Blood Cells

**DOI:** 10.3389/fimmu.2019.00160

**Published:** 2019-03-04

**Authors:** Myung-Hwa Jung, Verónica Chico, Sergio Ciordia, Maria Carmen Mena, Sung-Ju Jung, Maria Del Mar Ortega-Villaizan

**Affiliations:** ^1^Department of Aqualife Medicine, Chonnam National University, Gwangju, South Korea; ^2^IBMC-IDiBE, Universidad Miguel Hernandez, Elche, Spain; ^3^Unidad de Proteómica, Centro Nacional de Biotecnología (CSIC), Madrid, Spain

**Keywords:** rock bream, RBIV, red blood cells, erythrocyte, proteome, MHC class I, apoptosis, ISG15

## Abstract

Rock bream iridovirus (RBIV) causes severe mass mortality in Korean rock bream (*Oplegnathus fasciatus*) populations. To date, immune defense mechanisms of rock bream against RBIV are unclear. While red blood cells (RBCs) are known to be involved in the immune response against viral infections, the participation of rock bream RBCs in the immune response against RBIV has not been studied yet. In this study, we examined induction of the immune response in rock bream RBCs after RBIV infection. Each fish was injected with RBIV, and virus copy number in RBCs gradually increased from 4 days post-infection (dpi), peaking at 10 dpi. A total of 318 proteins were significantly regulated in RBCs from RBIV-infected individuals, 183 proteins were upregulated and 135 proteins were downregulated. Differentially upregulated proteins included those involved in cellular amino acid metabolic processes, cellular detoxification, snRNP assembly, and the spliceosome. Remarkably, the MHC class I-related protein pathway was upregulated during RBIV infection. Simultaneously, the regulation of apoptosis-related proteins, including caspase-6 (CASP6), caspase-9 (CASP9), Fas cell surface death receptor (FAS), desmoplakin (DSP), and p21 (RAC1)-activated kinase 2 (PAK2) changed with RBIV infection. Interestingly, the expression of genes within the ISG15 antiviral mechanism-related pathway, including filamin B (FLNB), interferon regulatory factor 3 (IRF3), nucleoporin 35 (NUP35), tripartite motif-containing 25 (TRIM25), and karyopherin subunit alpha 3 (KPNA3) were downregulated in RBCs from RBIV-infected individuals. Overall, these findings contribute to the understanding of RBIV pathogenesis and host interaction.

## Introduction

Rock bream iridovirus (RBIV) is a dsDNA virus that belongs to family Iridoviridae, genus *Megalocytivirus* (1). This virus causes severe mass mortality in Korean rock bream (*Oplegnathus fasciatus*) populations. RBIV was first reported in the summer of 1998 in southern coastal areas of Korea (2). Since then, high mortality resulting from RBIV occurs every year, causing important economic losses in rock bream aquaculture. RBIV is known to cause strong pathogenicity in rock bream individuals (3–7). To date, the immune response of rock bream with RBIV infection remains unclear, although it represents an important aquaculture health concern. Therefore, it is necessary to further detail the immune response mechanisms underlying the RBIV infection process in rock bream. Over the years, a considerable number of studies have investigated the immune response of rock bream at both physiological and molecular levels by transcriptomic and microarray analyses (8, 9). Recently, an increasing number of studies have been focused on the transcriptional immune responses of rock bream against RBIV (10–15). However, most have focused on kidney-mediated immune responses to determine the pathways responsible for fish mortality or survivability. Therefore, evaluation of the immune response or immune defense mechanisms in different organs is useful for the understanding host-RBIV interactions.

In contrast to mammalian red blood cells (RBCs) or erythrocytes, which lack a cell nucleus and organelles (16), nonmammalian RBCs are nucleated and contain organelles in their cytoplasm (17). Although the main physiological role for RBCs is the transportation of respiratory gases, their role in the antiviral response has recently been uncovered (18). Importantly, teleost RBCs can induce toll-like receptor (TLR) and peptidoglycan recognition protein (PGRP) receptor families (19), pathogen presentation to macrophages (20), and cytokine or interferon production (21–25). In addition, transcriptomic and proteomic studies of rainbow trout (*Oncorhynchus mykiss*) showed that nucleated RBCs contribute to several immune functions such as antigen presentation, leukocyte activation or immune cytokine production (26, 27).

To date, the impact of RBIV on rock bream RBCs in the global fish immune response has not been studied yet. In the present study, we aimed to investigate the differentially expressed proteins (DEPs) in rock bream RBCs upon RBIV *in vivo* infection in order to understand the molecular contribution of this cell type in the fish immune response against RBIV infection. Proteomic profiling of RBCs from RBIV-infected fish revealed upregulation of apoptosis, antigen processing, and presentation of peptide antigen via MHC class I (MHC-I) pathways. However, the ISG15 antiviral mechanism pathway appeared to be downregulated.

## Materials and Methods

### Isolation of RBIV

RBIV was obtained from naturally infected rock bream individuals as previously described (11). RBIV major capsid protein (MCP) gene copy number was quantified from supernatant preparations by quantitative real-time polymerase chain reaction (RT-qPCR). Virus titer was calculated as 1.1 × 10^7^/100 μL MCP gene copies. Although some studies have demonstrated the use of cell lines to culture *Megalocytivirus* (28, 29), RBIV does not replicate well in *in vitro* cell culture conditions, so the TCID_50_ method was not used in this study.

### Quantification of RBIV Viral Copy Number

RBIV-free rock bream individuals were obtained from a local farm. Thirty fish (11.2 ± 1.2 cm, 28.1 ± 3.2 g) were maintained at 23°C in an aquarium containing 250 L of UV-treated seawater. Fish were injected intraperitoneally (i.p.) with RBIV (100 μL/fish, 1.1 × 10^7^ MCP gene copies) or phosphate-buffered saline (PBS) (100 μL/fish) as a control. Blood (200 μL/fish) and organs (spleen, kidney, and liver) were collected from RBIV-infected rock bream individuals at 1, 2, 4, 7, and 10 days post infection (dpi) (4 fish per time point). RBCs were isolated from blood (100 μL/fish) and purified by 2 consecutive density gradient centrifugations (7,206 *g*, Ficoll 1.007, Sigma-Aldrich). For RBIV copy number analysis, genomic DNA was isolated from the RBCs, blood, spleen, kidney, and liver of each fish using High Pure PCR Template Preparation Kit (Roche) following standard protocol. A standard curve was generated to determine RBIV MCP gene copy number by RT-qPCR as described previously (11). Virus copy number was determined from 100 μL of total genomic DNA. Statistical analyses were performed using GraphPad Prism software version 5.0 (GraphPad Software, USA). One-way analysis of variance (ANOVA) was performed between conditions, with Tukey's multiple comparison test. *P* < 0.05 were considered to indicate statistical significance.

### Experimental Infection for RBC Proteomic Analysis

Fish (11.0 ± 0.8 cm, 29.3 ± 4.7 g) were randomly divided into two groups (20 fish per group): a virus-injected group and a PBS-injected group. The experimental group was injected i.p. with RBIV (100 μL/fish) containing 1.1 × 10^7^ MCP gene copies, and the control group was injected i.p. with PBS (100 μL/fish). Each group of fish were maintained at 23°C in the aquarium containing 250 L of UV-treated seawater. Blood (100 μL/fish) was collected from 8 fish at 7 dpi. Then, RBCs were purified by 2 consecutive density gradient centrifugations (7,206 *g*, Ficoll 1.007, Sigma-Aldrich). All rock bream experiments were carried out in strict accordance with the recommendations of the Institutional Animal Care and Use Committee of Chonnam National University (permit number: CNU IACUC-YS-2015-4).

### Proteomic Analysis

Ficoll-purified RBCs from 5 fish in each group were pelletized by centrifugation (1,600 rpm). The cell pellet was washed with PBS, digested, cleaned-up/desalted, and pooled for each group (2 control groups and 2 RBIV-infected fish groups). Then, samples were subjected to liquid chromatography and mass spectrometry analysis (LC-MS) as previously described (26), except that the Pierce High pH Reversed-Phase Peptide Fractionation Kit (Thermo Fisher Scientific, Inc.) was used and 3 peptide fractions were collected. Progenesis QI v4.0 (Nonlinear Dynamics, Newcastle, UK) was used for protein differential expression analysis according to “between-subject design.” Log_2_ peptide ratios followed a normal distribution that was fitted using least squares regression. Mean and standard deviation values were derived from Gaussian fit and were used to estimate *P*-values and false discovery rates (FDRs). The confidence interval for protein identification was set to ≥95% (*P* ≤ 0.05). Only proteins having ≥2 quantitated peptides were considered. Peptides with an individual ion score above the 1% FDR threshold were considered correctly identified.

### Pathway Enrichment Analysis

DEP pathway enrichment analysis was performed using ClueGO (30), CluePedia (31), and Cytoscape (32). The GO Biological Process, GO Immune Process, Kegg, Reactome, and Wikipathways databases were used. A *P* ≤ 0.05 and Kappa score of 0.4 were used as threshold values. Proteins were identified by sequence homology with *Homo sapiens* using Blast2GO version 4.1.9 (33).

### Quantitative Real-Time PCR Analysis of Gene Expression

For immune gene expression analysis, total RNA was extracted from RBCs using RNAiso Plus reagent (TaKaRa) following standard protocol. Total RNA was treated with DNase I (TaKaRa) and reverse transcribed using a ReverTra Ace qPCR RT Kit (Toyobo) according to manufacturer's protocol. Real-time PCR was carried out in an Exicycler 96 Real-Time Quantitative Thermal Block (Bioneer) using an AccuPre® 2x Greenstar qPCR Master Mix (Bioneer) as described previously (11). Each assay was performed in duplicate using β-actin genes as the endogenous control. The primers used are listed in Table 1. Relative gene expression was determined by the 2^−ΔΔ*Ct*^ method (34). Statistical analyses were performed using GraphPad Prism software. Unpaired *T*-tests were performed between conditions. *P* < 0.05 were considered to indicate statistical significance. Data are represented as mean ± standard deviation.

**Table 1:** List of primers used.

**Name**	**Sequence**	**Accession number**
β-actin	F CAGGGAGAAGATGACCCAGA R CATAGATGGGCACTGTGTGG	FJ975145
MCP	F GTGTCTAAAGGGACTGAACATCG R CCCTCAAACGTTACTGGATACTG	AY849394
IRF3	F TGGGAGTAACCCTTATGTCCTG R CTTCCTCGTCTGTTCCTTCTTG	KF267453.1
MHC class I	F AGATTACTGGGAAAAAGGCACA R TCATTCGTTTCATCAGGATGTC	KC193602
Fas	F GTTTCGTGCGTCGTTTATCA R CAAACCTGCAGCACACAGACA	AB619804
Caspase 9	F TCTTGGAGAGACACCCAGTCG R GCCCTTTTGCAGAGTTTTGG	KF501038

## Results

### RBIV Levels in Rock Bream RBCs

**Figure 1:**
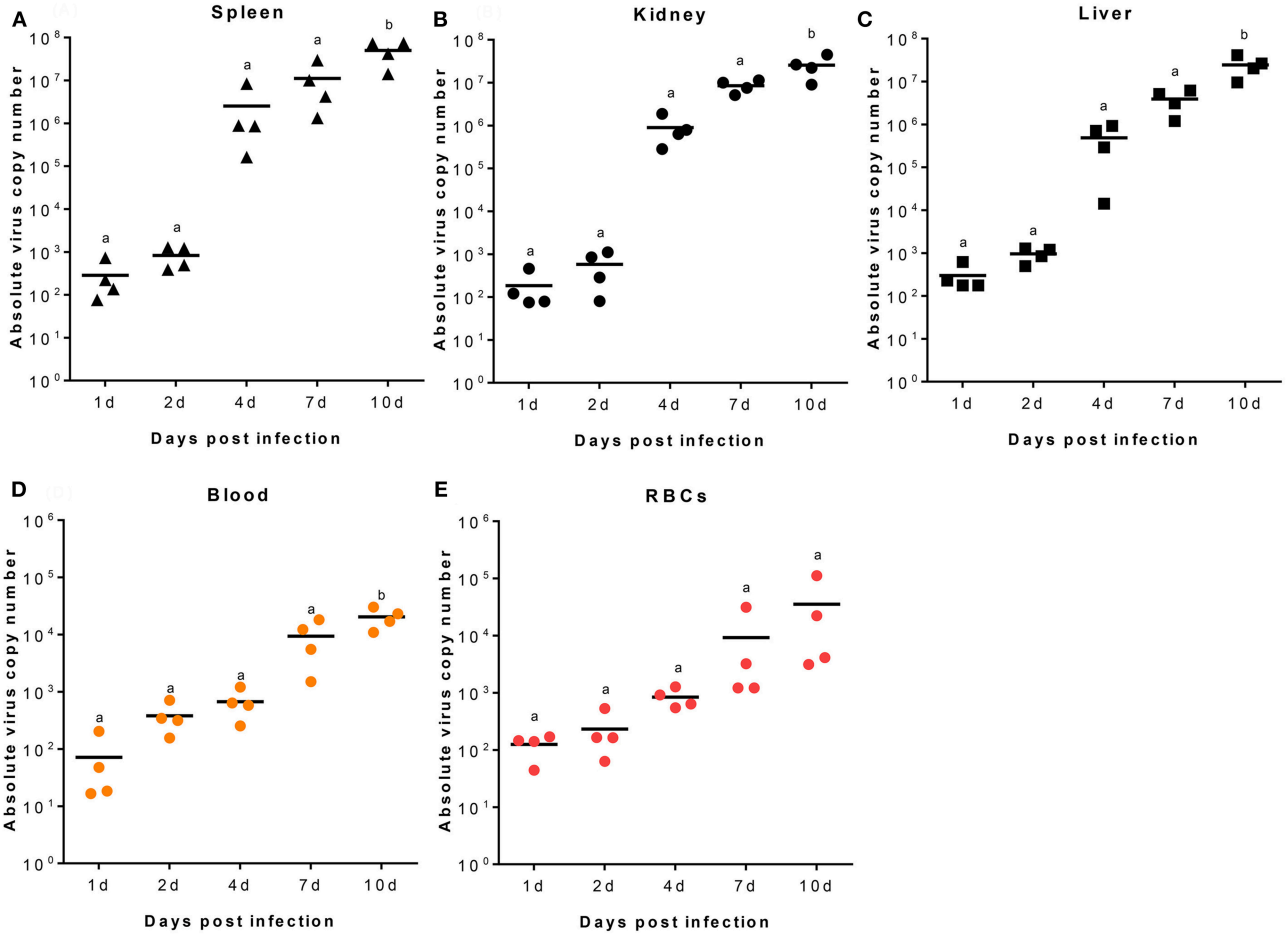
RBIV MCP gene copy number in different rock bream organs. Fish i.p. injected with RBIV (1.1 × 10^7^) were maintained at 23°C. Virus copy number in spleen **(A)**, kidney **(B)**, liver **(C)**, blood **(D)**, and RBCs **(E)** were analyzed at 1, 2, 4, 7, and 10 days post infection (dpi). One-way analysis of variance (ANOVA) was performed between conditions, with Tukey's multiple comparison test. Different superscript letters denote significant differences (*P* < 0.05). a≠ b. Data are represented as individual values. Line represents mean value.

RBIV copy number was quantified in RBC, blood, spleen, kidney, and liver samples. At 2, 4, 7, and 10 dpi, increased viral copy numbers were observed in the spleen, kidney, and liver. The maximum copy number for all samples was reached at 10 dpi (average value of 4.99 × 10^7^ in the spleen, 2.56 × 10^7^ in the kidney, and 2.44 × 10^7^/100 μL in the liver) (Figures 1A–C).

In blood samples, the viral transcription level was 7.16 × 10^1^/100 μL at 1 dpi, gradually increased to 3.81 × 10^2^/100 μL at 2 dpi, and reached maximum values of 9.36 × 10^3^/100 μL at 7 dpi and 2.04 × 10^4^/100 μL at 10 dpi (Figure 1D). In Ficoll-purified RBCs from fish at 1, 2, 4, 7, and 10 dpi, virus copy numbers gradually increased with time; the average number of virus copies was 1.25 × 10^2^, 2.31 × 10^2^, 8.42 × 10^2^, 9.22 × 10^3^, and 3.54 × 10^4^/100 μL, respectively (Figure 1E).

### Protein Profiling of RBCs From RBIV-Infected Rock Bream

**Figure 2:**
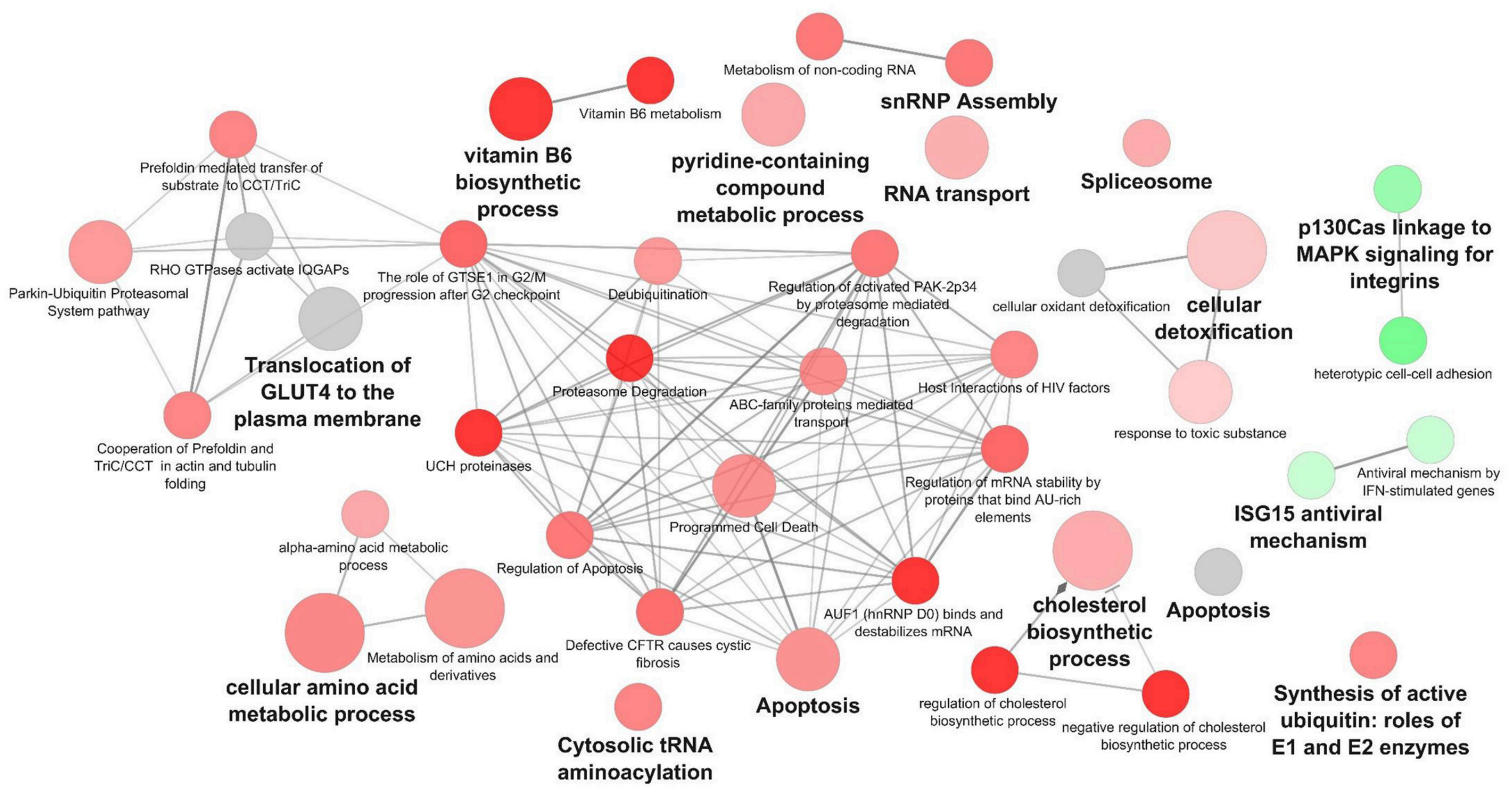
Cytoscape network analysis of differentially expressed protein (DEPs) in RBCs from RBIV-infected rock bream. DEPs in RBCs from RBIV-infected rock bream at 7 dpi, with −1.5 < log_2_FC < 1.5 and FDR *P* < 0.001. Overrepresented terms were identified by the Cytoscape ClueGo app, with GO Biological Process, Kegg, Reactome, and Wikipathways term databases. Red circles indicate upregulated/overrepresented terms, and green circles indicate downregulated/overrepresented terms. Gray circles indicate unspecific regulation. Color intensity represents the degree of overrepresentation.

**Figure 3:**
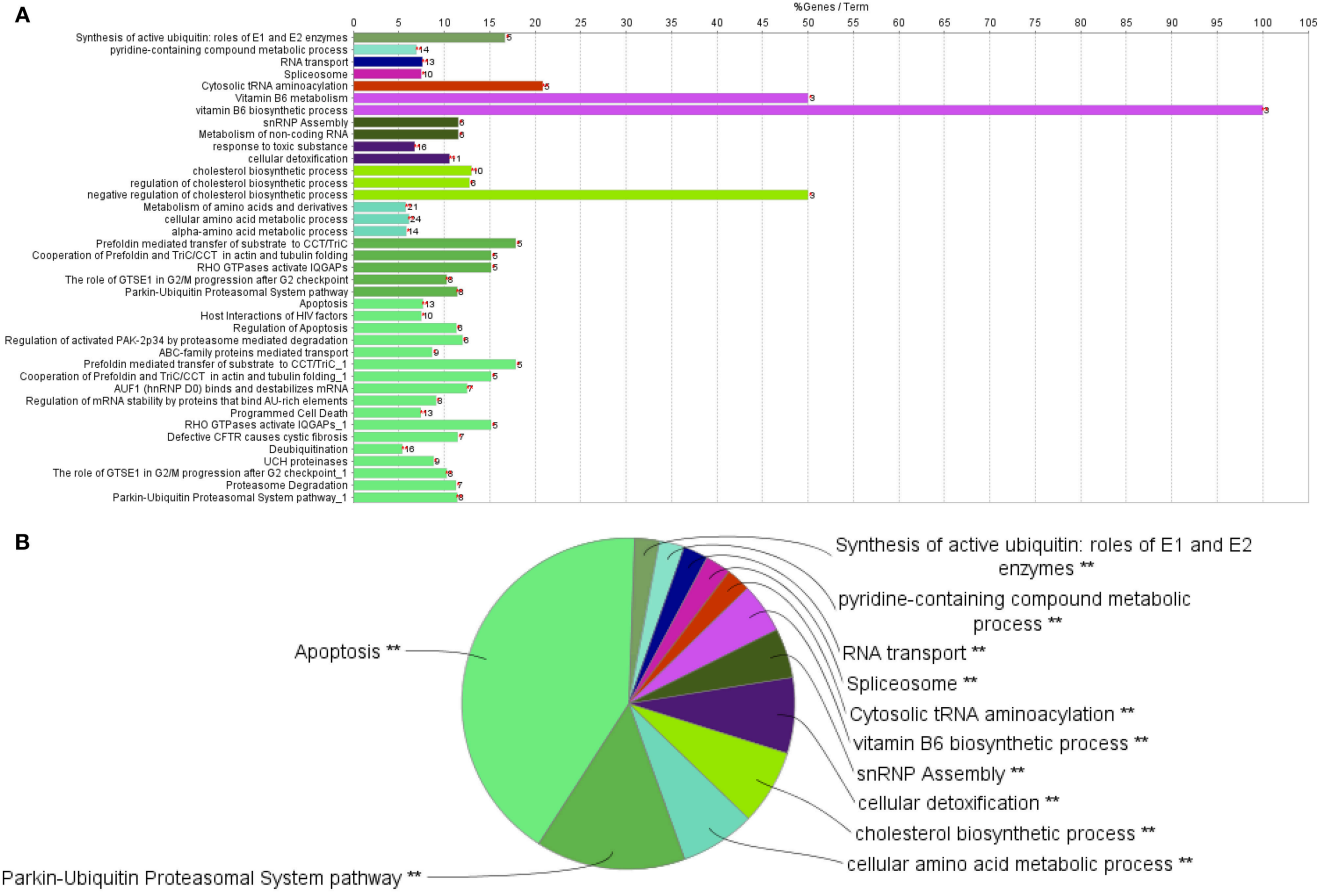
Upregulated functional pathways in the proteome profile of RBIV-infected RBCs. Upregulated/overrepresented terms in DEPs of RBCs from RBIV-infected rock bream at 7 dpi, with −1.5 < log_2_FC < 1.5 and FDR *P* < 0.001. **(A)** Bar graph and **(B)** multilevel pie chart. Overrepresented terms were identified by the Cytoscape ClueGo app, with GO Biological Process, Kegg, Reactome, and Wikipathways term databases. Asterisks denote GO-term significance (*P < 0.05 and **P < 0.01).

**Figure 4:**
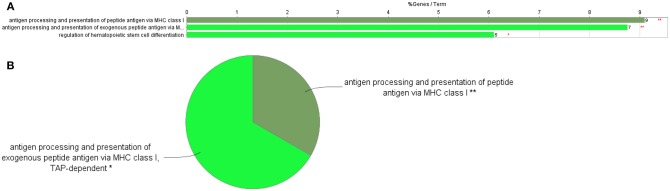
GO Immune System Process terms in the proteome profile of RBIV-infected RBCs. Upregulated/overrepresented terms in DEPs of RBCs from RBIV-infected rock bream at 7 dpi, with −1.5<log_2_FC<1.5 and FDR *P* < 0.001. **(A)** Bar graph and **(B)** multilevel pie chart. Overrepresented terms were identified by the Cytoscape ClueGo app with the GO Immune System Process database. Asterisks denote GO-term significance (**P < 0.01).

**Figure 5:**
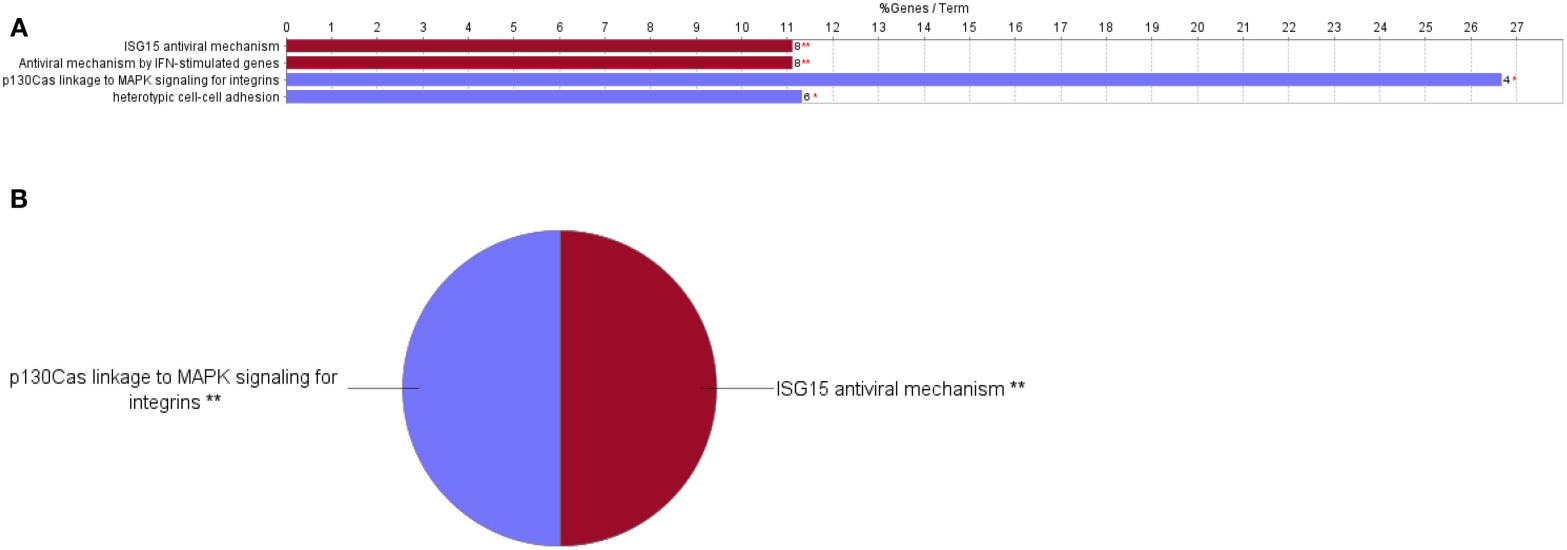
Downregulated functional pathways in the proteome profile of RBIV-infected RBCs. Downregulated/overrepresented terms in DEPs of RBCs from RBIV-infected rock bream at 7 dpi, with −1.5 < log_2_FC < 1.5 and FDR *P* < 0.001. **(A)** Bar graph and **(B)** multilevel pie chart. Overrepresented terms were identified by the Cytoscape ClueGo app, with the GO Biological Process, Kegg, Reactome, and Wikipathways databases. Asterisks denote GO-term significance (*P < 0.05 and **P < 0.01).

**Figure 6:**
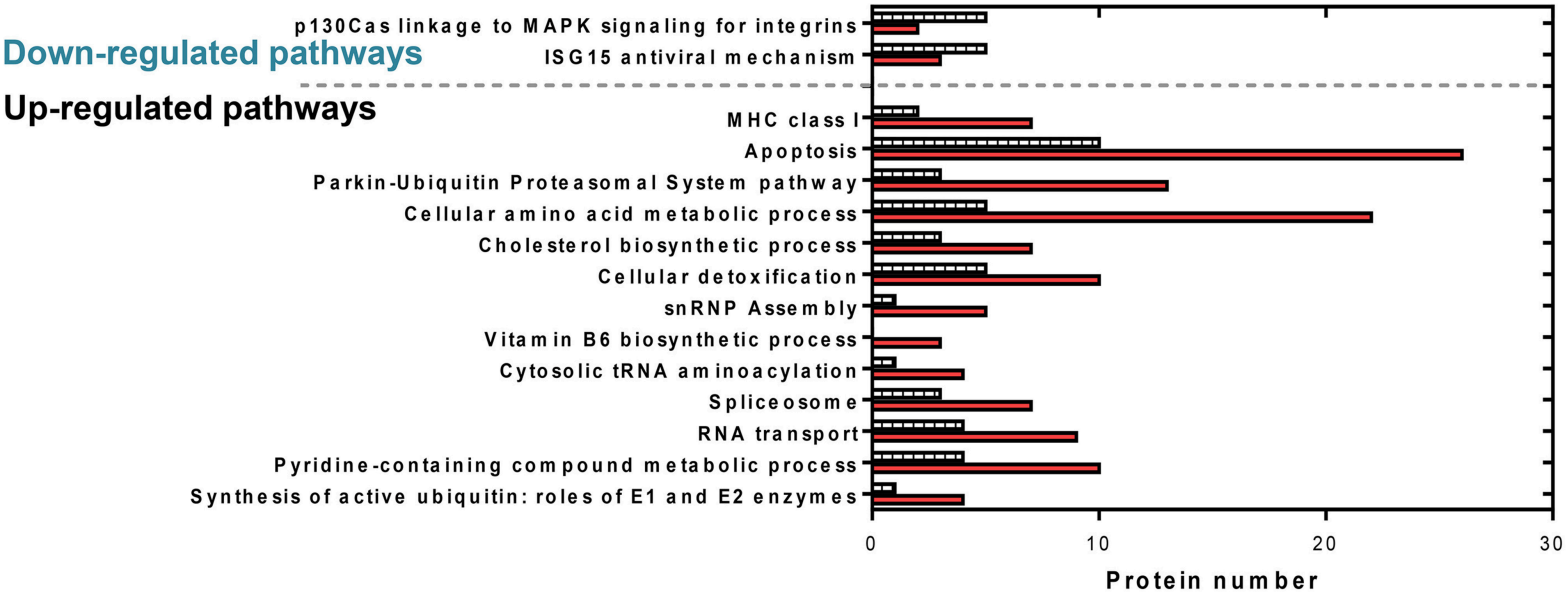
Comparative protein levels in upregulated and downregulated overrepresented pathways in RBCs from RBIV-infected rock bream. Data represent the number of proteins represented in each pathway. Red bars indicate upregulated proteins and dashed bars indicate downregulated proteins.

Cytoscape pathway enrichment analysis was performed in order to evaluate the functional pathways involved in the response of rock bream RBCs to RBIV (Figure 2). Proteins with a FDR < 0.001 and−1.5>log_2_ Fold Change (FC)>1.5 were selected for functional network analysis. A total of 318 proteins were differentially regulated at a significant level in RBCs from RBIV-infected individuals: 183 proteins were upregulated and 135 were downregulated. Upregulated pathways were categorized into 13 main categories, while downregulated pathways were categorized into 2 (Figures 2–6 and Tables 2–4). Within upregulated pathways, proteins were involved in synthesis of active ubiquitin, E1 and E2 enzymatic roles, pyridine-containing compound metabolic processes, RNA transport, the spliceosome, cytosolic tRNA aminoacylation, the vitamin B6 biosynthetic process, snRNP assembly, cellular detoxification, the cholesterol biosynthetic process, the cellular amino acid metabolic process, the Parkin-Ubiquitin proteasomal system pathway, apoptosis, and antigen processing and presentation of peptide antigen via MHC class I (Figures 2–4 and Tables 2, 3). Within downregulated pathways, proteins were mainly involved in the ISG15 antiviral mechanism and p130Cas linkage to MAPK signaling for integrins (Figures 2, 5, 6 and Table 4).

**Table 2:** List of upregulated pathways in RBCs from RBIV-infected rock bream.

**Category**	**Accession**	**Protein name**	**Protein description**	**Log_**2**_FC**
Synthesis of active ubiquitin: roles of E1 and E2 enzymes	A0A096M453	UCHL3	Ubiquitin C-terminal hydrolase L3	+4.54169
	A0A060YC09	UBE2L3	Ubiquitin conjugating enzyme E2 L3	+3.28977
	E7EXC7	USP9X	Ubiquitin specific peptidase 9 X-linked	+1.86819
	A0A1A8BMW9	USP5	Ubiquitin specific peptidase 5	+1.73518
	A0A1A7XFZ1	UBA6	Ubiquitin like modifier activating enzyme 6	−5.67014
Pyridine-containing compound metabolic process	A0A0P7UQB0	NUP98	Nucleoporin 98	+6.56510
	A0A060W490	PNPO	Pyridoxamine 5′-phosphate oxidase	+6.54472
	A0A1A8DQA8	PHGDH	Phosphoglycerate dehydrogenase	+5.96297
	A0A060X3S4	PDXK	Pyridoxal kinase	+3.52413
	A0A060X2R3	NUP93	Nucleoporin 93	+3.41500
	A0A023UJE3	ENO1	Enolase 1	+2.47019
	A0A060YZP7	MPC2	Mitochondrial pyruvate carrier 2	+2.35431
	A0A087XLW0	PGAM1	Phosphoglycerate mutase 1	+2.21249
	J3QRQ2	DCXR	Dicarbonyl and L-xylulose reductase	+1.89013
	A0A087Y0K3	PSAT1	Phosphoserine aminotransferase 1	+1.65664
	A0A087Y968	TPI1	Triosephosphate isomerase 1	−2.53038
	H3CAN5	GALK1	Galactokinase 1	−3.00322
	A0A146MRI7	NUP35	Nucleoporin 35	−6.06319
	A0A1A7XVE8	MDH1	Malate dehydrogenase 1	−7.96449
RNA transport	A0A0P7UQB0	NUP98	Nucleoporin 98	+6.56510
	A0A146RA28	EIF5B	Eukaryotic translation initiation factor 5B	+4.94553
	A0A087XQU0	PYM1	PYM homolog 1, exon junction complex associated factor	+4.00632
	H2MNB4	EIF2B3	Eukaryotic translation initiation factor 2B subunit gamma	+3.75534
	A0A060X2R3	NUP93	Nucleoporin 93	+3.41500
	C3KH96	RBM8	RNA binding motif protein 8A	+3.16004
	A0A060WH91	PABPC1	Poly(A) binding protein cytoplasmic 1	+2.51108
	A0A1A7XKU0	RANGAP1	Ran GTPase activating protein 1	+2.40663
	A0A087XK21	TRNT1	tRNA nucleotidyl transferase 1	+1.52564
	A0A087XJ99	EIF3I	Eukaryotic translation initiation factor 3 subunit I	−2.81793
	A0A060XCL3	ALYREF	Aly/REF export factor	−3.15159
	H2LP66	EIF3J	Eukaryotic translation initiation factor 3 subunit J	−4.12793
	A0A146MRI7	NUP35	Nucleoporin 35	−6.06319
Spliceosome	A0A0P7XD74	SNRPF	Small nuclear ribonucleoprotein polypeptide F	+8.79320
	A0A087Y346	SNRPD1	Small nuclear ribonucleoprotein D1 polypeptide	+4.98734
	I3KZX4	LSM3	LSM3 homolog, U6 small nuclear RNA and mRNA degradation associated	+3.60900
	C3KH96	RBM8	RNA binding motif protein 8A	+3.16004
	A0A060XGY3	SF3A3	Splicing factor 3a subunit 3	+2.23314
	A0A1L3A6A6	HSPA8	Heat shock protein family A (Hsp70) member 8	+1.81502
	A0A0P7UL65	SNRPG	Small nuclear ribonucleoprotein polypeptide G	+1.70328
	A0A087Y0E9	PPIH	Peptidylprolyl isomerase H	−3.11189
	A0A060XCL3	ALYREF	Aly/REF export factor	−3.15159
	H2RJ37	SNRPA1	Small nuclear ribonucleoprotein polypeptide A'	−3.29532
Cytosolic tRNA aminoacylation	G3NSI9	FARSLA	Phenylalanyl-tRNA synthetase subunit alpha	+3.43435
	A0A1A7ZJC0	MARS	Methionyl-tRNA synthetase	+3.27543
	A0A087YJF0	EPRS	Glutamyl-prolyl-tRNA synthetase	+2.78295
	A0A060YC35	SARS	Seryl-tRNA synthetase	+2.61934
	A0A060WQF7	LARS	Leucyl-tRNA synthetase	−1.93372
	A0A060W490	PNPO	Pyridoxamine 5′-phosphate oxidase	+6.54472
	A0A060X3S4	PDXK	Pyridoxal kinase	+3.52413
	A0A087Y0K3	PSAT1	Phosphoserine aminotransferase 1	+1.65664
snRNP Assembly	A0A0P7XD74	SNRPF	Small nuclear ribonucleoprotein polypeptide F	+8.79320
	A0A0P7UQB0	NUP98	Nucleoporin 98	+6.56510
	A0A087Y346	SNRPD1	Small nuclear ribonucleoprotein D1 polypeptide	+4.98734
	A0A060X2R3	NUP93	Nucleoporin 93	+3.41500
	A0A0P7UL65	SNRPG	Small nuclear ribonucleoprotein polypeptide G	+1.70328
	A0A146MRI7	NUP35	Nucleoporin 35	−6.06319
Cellular detoxification	A0A087YGW8	CLIC2	Chloride intracellular channel 2	+6.00740
	H2RV41	GSTM3	Glutathione S-transferase mu 3	+5.94070
	I3IV50	FAS	Fas cell surface death receptor	+5.88751
	W5KQL6	APOE	Apolipoprotein E	+4.62692
	A0A0S7HP87	FAM213B	Family with sequence similarity 213 member B	+4.13534
	B9MSR2	SOD1	Superoxide dismutase 1	+2.53220
	A0A087X9L9	TXNRD3	Thioredoxin reductase 3	+2.07657
	A0A060VRY4	XPA	XPA, DNA damage recognition and repair factor	+1.76996
	A0A087YMH6	ADH5	Alcohol dehydrogenase 5 (class III), chi polypeptide	+1.57015
	W5NF82	NEFL	Neurofilament light	+1.50524
	A0A087YDB9	TRPM6	Transient receptor potential cation channel subfamily M member 6	−2.90258
	B3VTP4	APOA4	Apolipoprotein A4	−3.50052
	A0A087WSW9	TXNRD1	Thioredoxin reductase 1	−3.51362
	C9DTM6	EPX	Eosinophil peroxidase	−5.96073
	A0A0F8BVI8	MPO	Myeloperoxidase	−5.96073
Cholesterol biosynthetic process	W5KQL6	APOE	Apolipoprotein E	+4.62692
	W5NG17	GGPS1	Geranylgeranyl diphosphate synthase 1	+3.68607
	A0A0S7LJM9	CNBP	CCHC-type zinc finger nucleic acid binding protein	+3.65477
	A0A060X0E0	ERLIN2	ER lipid raft associated 2	+3.09663
	A0A060WK05	PMVK	Phosphomevalonate kinase	+3.00278
	C1BJ00	VDAC2	Voltage dependent anion channel 2	+2.784311
	B9MSR2	SOD1	Superoxide dismutase 1	+2.53220
	B3VTP4	APOA4	Apolipoprotein A4	−3.50052
	C1BKM7	APOA1	Apolipoprotein A1	−3.58118
	I6QFY3	CFTR	Cystic fibrosis transmembrane conductance regulator	−3.85295
Cellular amino acid metabolic process	A0A146NIL6	HNMT	Histamine N-methyltransferase	+7.33475
	Q19A30	ALDH9A1	Aldehyde dehydrogenase 9 family member A1	+7.08477
	H2M1L3	GCLC	Glutamate-cysteine ligase catalytic subunit	+7.05565
	A0A1A8DQA8	PHGDH	Phosphoglycerate dehydrogenase	+5.96297
	A0A087YCZ2	SBDS	SBDS, ribosome maturation factor	+5.38388
	H2SS02	PYCR3	Pyrroline-5-carboxylate reductase 3	+4.13757
	A0A0P7USQ3	PSMD11	Proteasome 26S subunit, non-ATPase 11	+3.83617
	W5UAL8	GSS	Glutathione synthetase	+3.49635
	A0A087X9P9	RPS28	Ribosomal protein S28	+3.46599
	G3NSI9	FARSLA	Phenylalanyl-tRNA synthetase subunit alpha	+3.43435
	A0A1A7ZJC0	MARS	Methionyl-tRNA synthetase	+3.27543
	A0A147AHI6	PSMB6	Proteasome subunit beta 6	+3.23477
	Q66HW0	COASY	Coenzyme A synthase	+2.88808
	A0A087YJF0	EPRS	Glutamyl-prolyl-tRNA synthetase	+2.78295
	A0A087WUL2	PSMB3	Proteasome subunit beta 3	+2.74293
	A0A060YC35	SARS	Seryl-tRNA synthetase	+2.61934
	H2VBD9	PSMD5	Proteasome 26S subunit, non-ATPase 5	+2.46929
	A0A060YZH5	RPS21	Ribosomal protein S21	+2.03250
	A0A0N8K350	ARG2	Arginase 2	+1.90666
	H2MN42	NIT2	Nitrilase family member 2	+1.87753
	Q45VN8	PSMB4	Proteasome subunit beta 4	+1.84703
	A0A087Y0K3	PSAT1	Phosphoserine aminotransferase 1	+1.65664
	A0A087XKC8	ALDH4A1	Aldehyde dehydrogenase 4 family member A1	−1.60365
	A0A0F8C9G0	AASDHPPT	Aminoadipate-semialdehyde dehydrogenase-phosphopantetheinyl transferase	−1.8438
	W5M476	SARDH	Sarcosine dehydrogenase	−1.86083
	A0A060WQF7	LARS	Leucyl-tRNA synthetase	−1.93372
	A0A060Z3T7	MRI1	Methylthioribose-1-phosphate isomerase 1	−2.64492
	A0A087WSW9	TXNRD1	Thioredoxin reductase 1	−3.51362
Parkin-ubiquitin proteasomal system pathway	A0A146UQZ0	CCT3	Chaperonin containing TCP1 subunit 3	+4.20768
	A0A0P7USQ3	PSMD11	Proteasome 26S subunit, non-ATPase 11	+3.83617
	A0A060YC09	UBE2L3	Ubiquitin conjugating enzyme E2 L3	+3.28977
	A0A147AHI6	PSMB6	Proteasome subunit beta 6	+3.23477
	A0A146VFH4	TUBA4A	Tubulin alpha-4A chain	+2.86588
	A0A060WLR9	TUBA3C	Tubulin alpha 3c	+2.86588
	A0A087WUL2	PSMB3	Proteasome subunit beta 3	+2.74293
	H2VBD9	PSMD5	Proteasome 26S subunit, non-ATPase 5	+2.46929
	Q45VN8	PSMB4	Proteasome subunit beta 4	+1.84703
	A0A146PU69	ACTB	Actin beta	+1.83645
	A0A1L3A6A6	HSPA8	Heat shock protein family A (Hsp70) member 8	+1.81502
	A0A189JAM4	TUBA1C	Tubulin alpha 1c	−2.55283
	H6QXT0	CASP1	Caspase 1	−2.90548
	F2Z2E2	IQGAP3	IQ motif containing GTPase activating protein 3	−4.82941
Apoptosis	A0A0P7UQB0	NUP98	Nucleoporin 98	+6.56510
	I3IV50	FAS	Fas cell surface death receptor	+5.88751
	A0A060WPW9	RUVBL1	RuvB like AAA ATPase 1	+5.68115
	A0A060X986	CASP9	Caspase 9	+5.34643
	A0A096M453	UCHL3	Ubiquitin C-terminal hydrolase L3	+4.54169
	W5LA34	ABCB1	ATP binding cassette subfamily B member 1	+4.23220
	A0A146UQZ0	CCT3	Chaperonin containing TCP1 subunit 3	+4.20768
	A0A0P7USQ3	PSMD11	Proteasome 26S subunit, non-ATPase 11	+3.83617
	A0A060X2R3	NUP93	Nucleoporin 93	+3.41500
	A0A060YC09	UBE2L3	Ubiquitin conjugating enzyme E2 L3	+3.28977
	A0A147AHI6	PSMB6	Proteasome subunit beta 6	+3.23477
	A0A060X0E0	ERLIN2	ER lipid raft associated 2	+3.09663
	C1BJ00	VDAC2	Voltage dependent anion channel 2	+2.78431
	A0A087WUL2	PSMB3	Proteasome subunit beta 3	+2.74293
	A0A060XWP8	RPN2	Ribophorin II	+2.51942
	A0A060WH91	PABPC1	Poly(A) binding protein cytoplasmic 1	+2.51108
	H2VBD9	PSMD5	Proteasome 26S subunit, non-ATPase 5	+2.46929
	A0A087XG68	HMGB2	High mobility group box 2	+2.41814
	A0A1A7XKU0	RANGAP1	Ran GTPase activating protein 1	+2.40663
	A0A146RM67	DSP	Desmoplakin	+2.25958
	A0A060VUK9	ACTL6A	Actin like 6A	+1.92039
	E7EXC7	USP9X	Ubiquitin specific peptidase 9 X-linked	+1.86819
	Q45VN8	PSMB4	Proteasome subunit beta 4	+1.84703
	A0A1L3A6A6	HSPA8	Heat shock protein family A (Hsp70) member 8	+1.81502
	A0A1A8BMW9	USP5	Ubiquitin specific peptidase 5	+1.73518
	H2MXM9	CASP6	Caspase 6	+1.65460
	A0A060W5L7	USP47	Ubiquitin specific peptidase 47	−1.85717
	A0A1A8GUB0	YWHAB	Tyrosine 3-monooxygenase/tryptophan 5-monooxygenase activation protein beta	−2.17782
	A0A060WMK5	PAK2	p21 (RAC1) activated kinase 2	−2.39132
	G3NDG3	PLEC	Plectin	−3.20510
	G3NRU2	RNF146	Ring finger protein 146	−3.25047
	C1BKM7	APOA1	Apolipoprotein A1	−3.58118
	I6QFY3	CFTR	Cystic fibrosis transmembrane conductance regulator	−3.85294
	F2Z2E2	IQGAP3	IQ motif containing GTPase activating protein 3	−4.82941
	X1WEE8	TRIM25	Tripartite motif containing 25	−5.61605
	A0A146MRI7	NUP35	Nucleoporin 35	−6.06319

**Table 3:** List of identified proteins related to antigen processing and presentation of peptide antigen via MHC class I.

**Category**	**Accession**	**Protein name**	**Protein description**	**Log_**2**_FC**
Antigen processing and presentation of peptide antigen via MHC class I	A0A146MHT9	MR1	Major histocompatibility complex, class I-related	+4.08719
	A0A0P7USQ3	PSMD11	Proteasome 26S subunit, non-ATPase 11	+3.83617
	Q5SRD4	TAP2	Transporter 2, ATP binding cassette subfamily B member	+3.83464
	A0A147AHI6	PSMB6	Proteasome subunit beta 6	+3.23477
	A0A087WUL2	PSMB3	Proteasome subunit beta 3	+2.74293
	H2VBD9	PSMD5	Proteasome 26S subunit, non-ATPase 5	+2.46929
	Q45VN8	PSMB4	Proteasome subunit beta 4	+1.84703
	I3J5Y7	CANX	Calnexin	−1.55500
	A5A0E1	SNAP23	Synaptosome associated protein 23	−2.80077
Antigen processing and presentation of exogenous peptide antigen via MHC class I	A0A0P7USQ3	PSMD11	Proteasome 26S subunit, non-ATPase 11	+3.83617
	Q5SRD4	TAP2	Transporter 2, ATP binding cassette subfamily B member	+3.83464
	A0A147AHI6	PSMB6	Proteasome subunit beta 6	+3.23477
	A0A087WUL2	PSMB3	Proteasome subunit beta 3	+2.74293
	H2VBD9	PSMD5	Proteasome 26S subunit, non-ATPase 5	+2.46929
	Q45VN8	PSMB4	Proteasome subunit beta 4	+1.84703
	A5A0E1	SNAP23	Synaptosome associated protein 23	−2.80077

**Table 4:** List of downregulated pathways in RBCs from RBIV-infected rock bream.

**Category**	**Accession**	**Protein name**	**Protein description**	**Log_**2**_FC**
ISG15 antiviral mechanism	A0A0P7UQB0	NUP98	Nucleoporin 98	+6.56510
	A0A060X2R3	NUP93	Nucleoporin 93	+3.41500
	C7ATZ0	STAT1	Signal transducer and activator of transcription 1	+2.72893
	A0A060W790	KPNA3	Karyopherin subunit alpha 3	−1.55875
	A0A067ZTD7	IRF3	Interferon regulatory factor 3	−2.77578
	X1WEE8	TRIM25	Tripartite motif containing 25	−5.61605
	A0A087X811	FLNB	Filamin B	−5.77028
	A0A146MRI7	NUP35	Nucleoporin 35	−6.06319
p130Cas linkage to MAPK signaling for integrins	Q6PH06	CRK	CRK proto-oncogene, adaptor protein	+3.83669
	A0A146RM67	DSP	Desmoplakin	+2.25958
	A0A0F8ALN2	FGA	Fibrinogen alpha chain	−1.84245
	H2LW76	FGG	Fibrinogen gamma chain	−3.25828
	C1BKM7	APOA1	Apolipoprotein A1	−3.58117
	A0A087X4W0	FGB	Fibrinogen beta chain	−4.94492
	A0A0R4ICS1	ITGA4	Integrin subunit alpha 4	−5.35249

### Differentially Expressed Proteins Related to the Apoptosis Functional Pathway

A total of 36 apoptosis-related proteins were differentially regulated in RBCs from RBIV-infected individuals: 26 proteins were upregulated and 10 were downregulated (Figure 6). Among them, caspase-6 (CASP6), caspase-9 (CASP9), fas cell surface death receptor (FAS), and desmoplakin (DSP) were upregulated at 1.65, 5.35, 5.89, and 2.26 log_2_FC, respectively (Table 2). p21 (RAC1)-activated kinase 2 (PAK2) was downregulated at −2.39 log_2_FC (Table 2).

### Differentially Expressed Proteins Related to the Spliceosome and snRNP Assembly Functional Pathways

Ten spliceosome-related proteins were differentially regulated in RBCs from RBIV-infected individuals: 7 proteins were upregulated and 3 were downregulated (Figure 6 and Table 2). Moreover, 6 snRNP assembly-related proteins were differentially expressed: 5 proteins upregulated and 1 protein downregulated (Figure 6 and Table 2). Among upregulated proteins, the top-scored was small nuclear ribonucleoprotein polypeptide F (SNRPF), with 8.79 log_2_FC. In addition, small nuclear ribonucleoprotein D1 polypeptide (SNRPD1) and small nuclear ribonucleoprotein polypeptide G (SNRPG) were highly upregulated (Table 2).

### Differentially Expressed Proteins Related to Cellular Amino Acid Metabolic Processes and Cellular Detoxification Pathways

A total of 28 DEPs in RBCs from RBIV-infected individuals were involved in cellular amino acid metabolic processes, including 22 upregulated and 6 downregulated proteins (Figure 6 and Table 2). Among upregulated proteins, histamine N-methyltransferase (HNMT), aldehyde dehydrogenase 9 family member A1 (ALDH9A1), glutamate-cysteine ligase catalytic subunit (GCLC), phosphoglycerate dehydrogenase (PHGDH), ribosome maturation factor (SBDS), and pyrroline-5-carboxylate reductase 3 (PYCR3) were highly upregulated with log_2_FC of 7.33, 7.08, 7.06, 5.96, 5.38, and 4.14, respectively (Table 2).

Of the 15 DEPs involved in cellular detoxification, 10 were upregulated (from 1.50 to 6.94 log_2_FC) and 5 were downregulated (from −2.90 to −5.96 log_2_FC) (Table 2). Of note, upregulated proteins included antioxidant enzymes such as glutathione S-transferase mu 3 (GSTM3), superoxide dismutase 1 (SOD1), and thioredoxin reductase 3 (TXNRD3).

### Differentially Expressed Proteins Involved in Antigen Processing and Presentation of Peptide Antigen Via MHC Class I

Of 9 DEPs in RBCs from RBIV-infected individuals involved in antigen processing and presentation of peptide antigen via MHC class I (Figure 4), 7 were upregulated and 2 were downregulated (Figure 6 and Table 3). Among the upregulated proteins (with log_2_FC ranging from 1.85 to 4.08), were major histocompatibility complex class I-related protein (MR1), transporter 2 ATP binding cassette subfamily B member (TAP2), and 6 proteasome subunit proteins (proteasome 26S subunit non-ATPase 11 [PSMD11], proteasome subunit beta 6 [PSMB6], proteasome subunit beta 3 [PSMB3], proteasome 26S subunit non-ATPase 5 [PSMD5], and proteasome subunit beta 4 [PSMB4]).

### Differentially Expressed Proteins Involved in ISG15 Antiviral Mechanism Pathway

The interferon-stimulated gene 15 (ISG15) antiviral mechanism pathway appeared to be mainly downregulated in RBCs from RBIV-infected rock bream (Figure 5). Within this pathway, 3 proteins were upregulated (signal transducer and activator of transcription 1 [STAT1], nucleoporin 93 [NUP93], and nucleoporin 98 [NUP98], with log_2_FC ranging from 2.73 to 6.57), and 5 were downregulated (filamin B [FLNB], nucleoporin 35 [NUP35], interferon regulatory factor 3 [IRF3], tripartite motif containing 25 [TRIM25], and karyopherin subunit alpha 3 [KPNA3], with log_2_FC ranging from −1.56 to −6.06) (Figure 6 and Table 4).

### Validation of Representative Identified Proteins by Means of RT-qPCR

**Figure 7:**
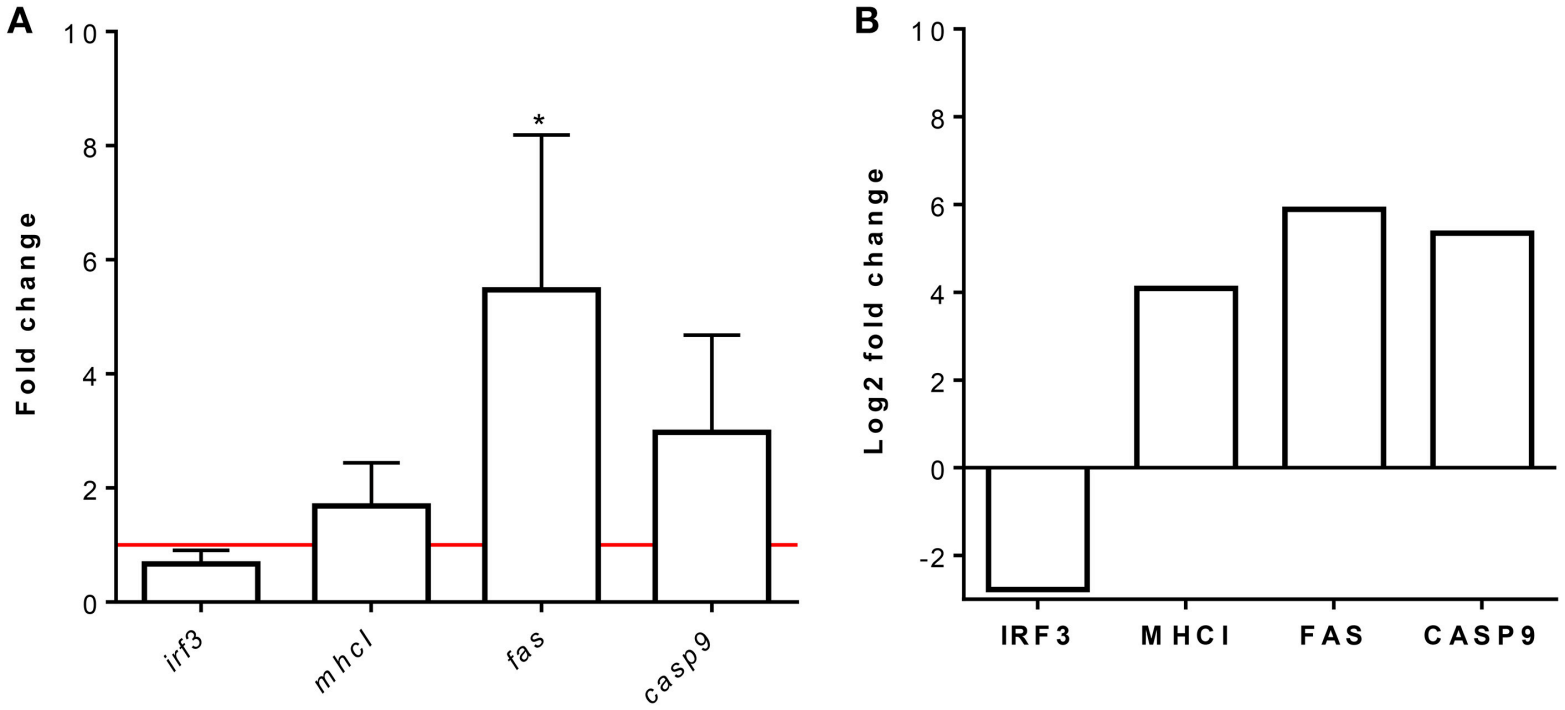
Relative mRNA and protein expression analysis of IRF3, MHCI, FAS, and CASP9. RBCs from RBIV-infected rock bream compared to PBS-injected rock bream (control). **(A)** Gene expression analysis, relative to control individuals (red line), evaluated by means of RT-qPCR. The β*-actin* gene was used as an endogenous control. Bars represent the mean ± standard deviation (SD) (*n* = 4 individuals). Unpaired *T*-tests were performed between conditions.*P < 0.05. **(B)** Quantitative protein expression values of selected proteins for pathway validation from proteomic analysis. Bars indicate log_2_FC value. FDR values are indicated in Supplementary Table S1.

Representative proteins were selected from each overrepresented pathway for validation at the transcriptional level. The *Fas* and *casp9* genes were selected as representatives of the apoptosis pathway, the *mhcI* gene was selected as a representative of antigen processing and presentation of peptide antigens via MHCI, and the *irf3* gene was selected as a representative of the ISG15 antiviral mechanism. As shown in Figure 7, the expression levels of these proteins correlated with the RT-qPCR transcript levels.

## Discussion

In this study, we report relevant findings in which RBIV, an economically important virus in rock bream aquaculture production, induce an immune response in RBCs. The spleen is one of the major target organs for RBIV replication (2–4, 7). However, we found similarities in RBIV level patterns in the spleen, kidneys, liver, blood, and RBCs. RBIV copy numbers were not as high as in RBCs as in other organs. Nonetheless, RBIV time-dependent increments were found in rock bream blood or Ficoll-purified RBCs.

Previous microarray analyses of kidney samples from RBIV-infected rock bream have shown that hemoglobin (α and β) expression gradually decreased after RBIV replication reached its maximum levels (around 10^6^ to 10^7^/μL) at 20 to 25 dpi (unpublished data). In contrast, high levels of hemoglobin expression were observed at 70 dpi when low viral loads were detected (below 10^2^/μL) (unpublished data). On the other hand, rock bream individuals treated with poly (I:C) exhibited high expression levels of *irf3, isg15*, and protein kinase RNA-activated (*pkr*) genes in blood samples, whereas no significant upregulation was observed in the spleen or kidney (6). Furthermore, the highest *mhcI* constitutive gene expression was detected in the blood of rock bream compared to other tissues such as spleen or kidney (10). Together, these findings emphasize the importance of evaluating blood-mediated immune responses in rock bream against RBIV infection.

RBCs are the most common cell type in the blood, so understanding their immune response will be essential to identify future strategies for controlling RBIV infection. In the present study, we evaluated the proteome of RBCs from RBIV-infected rock bream. Among the upregulated proteins, the MHCI and apoptosis-related pathways were the most overrepresented in RBCs from RBIV-infected rock bream. MHCI plays a crucial role in the presentation of antigen peptides, which are produced by the degradation of intracellular pathogens. These antigen peptides then bind to MHCI molecules and are presented to CD8^+^ T lymphocytes to trigger cellular immune responses and induce the elimination of infected or apoptotic cells (35, 36). Apoptosis is a process of programmed cell death known to prevent the transmission of infection to uninfected healthy cells by killing infected cells (37). Cytotoxic lymphocytes (CTL) kill infected cells by 2 main pathways: i) releasing cytolytic granules such as pore-forming protein perforin and serine protease granzymes (38, 39) and ii) activating the caspase-dependent Fas ligand pathway (40, 41). In the present study, antigen processing and presentation of peptide antigen via MHCI was upregulated in RBCs from RBIV-infected rock bream. Simultaneously, FAS and CASP9, two proteins implicated in the caspase-dependent Fas ligand pathway, were upregulated in RBCs from RBIV-infected rock bream. Indeed, it has been reported that cytotoxic effector cells induce apoptosis in response to RBIV infection (11). In addition, perforin- and granzyme-related apoptosis initiation signals have been reported to be activated in the kidneys of RBIV-infected rock bream. However, the authors also reported that the Fas-induced, caspase-dependent apoptosis pathway was barely induced based on only slight increases in *fas, casp3, casp8*, and *casp9* gene expression (11, 13). Conversely, based on our proteomic results, both FAS and CASP9 proteins were upregulated in RBCs from RBIV-infected individuals, indicating that RBIV-activated apoptosis in rock bream RBCs could occur via the caspase-dependent Fas ligand pathway. These results could also suggest that apoptosis-related genes may be differently expressed in kidneys and RBCs. Similarly, we have previously reported that a myristoylated membrane protein (MMP)-based DNA vaccine administered to rock bream triggered differential expression of apoptosis-related genes (including perforin, granzyme, Fas, Fas ligand, and caspases) depending on the tissue analyzed (spleen, kidney, liver, or muscle) (42). In addition, we have observed that other proteins involved in promoting or inducing apoptosis, such as DSP, PAK2, and heat shock protein family A (Hsp70) member 8 (HSPA8) proteins, were highly upregulated in rock bream RBCs upon RBIV infection. The induction of both the antigen processing and presentation via MHCI pathway and the apoptosis-related pathway against RBIV infection may indicate that RBCs attempt to activate CTLs and subsequently trigger them to induce apoptosis by perforin and granzyme production, which are critical factors for the inhibition of RBIV replication (13). Separately, MHCI-induced apoptosis has been also reported during differentiation and activation of certain hematopoietic cells (43).

Surprisingly, in the present study, proteins related to the ISG15 antiviral mechanism such as IRF3, NUP35, and TRIM25 were downregulated in RBCs from RBIV-infected individuals. In general, the first line of defense against viral infection is based on type I interferon (IFN) expression (44). ISG15 is known to play an antiviral role against different viral pathogens [reviewed in (45)]. In fish, the IFN-related immune response, as well as ISG15-related proteins, are known to exhibit an inhibitory effect on viral infections (46–53). In our previous studies, we have found that *mx* gene expression upregulation occurs soon after viral infection and is maintained in the kidneys of RBIV-infected rock bream at least till 10 dpi (15). However, the expression of the *isg15* and *pkr* genes declined after 4 dpi. Therefore, type I IFN responses induced by RBIV infection seemed to be limited in time and were not able to maintain antiviral responses at later stages, leading to fish mortality (15). Many viruses have developed strategies to counteract the antiviral activity of ISG15 (54). In orange-spotted grouper (*Epinephelus coioides*) spleen cell line (GS), ISG15 was not significantly upregulated by Singapore grouper iridovirus (SGIV) infection, while it was overexpressed by grouper nervous necrosis virus (GNNV) (45). Moreover, SGIV infection could downregulate the expression of ISG15, IFN and Mx previously induced by poly I:C, suggesting that SGIV was able to counteract the cellular interferon-mediated antiviral activity. In this regard, the authors also speculated that SGIV encoded proteins could play vital roles in preventing ISG15 activity during SGIV infection. To our knowledge, nothing is known about the interactions between RBIV proteins and host innate immune responses, especially those related to IFN or ISG15 pathways proteins. Therefore, in light of evidences, further studies are needed to elucidate RBIV interactions and/or counteracting effects on rock bream innate immune response.

Finally, pathways related to the spliceosome, snRNP assembly, cellular amino acid metabolic processes, and cellular detoxification were differentially regulated in RBCs from RBIV-infected rock bream. In the same way, previous investigations by Nombela et al. have reported the regulation of proteins related to spliceosomal complex and antioxidant/antiviral response in RBCs exposed *in vitro* to VHSV (23). However, how these mechanisms contribute to rock bream immune response to RBIV remains to be studied.

In summary, we have demonstrated that rock bream RBCs are able to generate a response to RBIV infection. This response was characterized by the upregulation of apoptosis-, MHCI, cellular detoxification-, and spliceosome-related pathways and the downregulation of ISG15 antiviral mechanisms. We have therefore identified novel target proteins in RBCs that will be valuable tools for future studies on the elucidation of RBIV-rock bream interaction mechanisms. These relevant findings will contribute to mitigate an economically important viral disease affecting rock bream aquaculture.

## Author Contributions

M-HJ performed experiments, analyzed data, and wrote the manuscript. VC performed experiments. SC and MM performed proteomic sequencing. MO-V conceived ideas, analyzed data, oversaw the research, and wrote the manuscript. VC and S-JJ contributed to the preparation of the manuscript.

### Conflict of Interest Statement

The authors declare that the research was conducted in the absence of any commercial or financial relationships that could be construed as a potential conflict of interest.
